# Impact of Formulation Choices on the Freeze-Drying of an Interleukin-6 Reference Material

**DOI:** 10.3389/fmolb.2022.868460

**Published:** 2022-07-04

**Authors:** Paul Matejtschuk, Christopher Bird, Ernest Ezeajughi, Kirsty MacLellan-Gibson, Meenu Wadhwa

**Affiliations:** ^1^ Analytical and Biological Sciences, NIBSC, Medicines and Healthcare Products Regulatory Agency, Potters Bar, United Kingdom; ^2^ Biotherapeutics, NIBSC, Medicines and Healthcare products Regulatory Agency, Potters Bar, United Kingdom

**Keywords:** freeze drying, formulation, interleukin-6, sodium chloride, scanning electron microscopy, differential scanning calorimetry

## Abstract

Formulation is critical to successful delivery of lyophilized biologics. We have compared the impact of buffer choice and the addition of sodium chloride (a formulant often viewed as unfavorable for freeze-drying applications) on the outcome of trial lyophilization of an interleukin-6 reference material. While phosphate buffer was a preferred choice and yielded well-formed cakes associated with fair recovery of biological activity, the resultant residual moisture content was high (2–4% w/w). By inclusion of isotonic levels of NaCl, the freeze-dried appearance and process were not impaired, but the residual moisture delivered was considerably reduced to levels <1% w/w. We postulate that this is due to the presence of a more open-cake structure and support this with evidence from thermal analysis and scanning electron microscopy. This work illustrates the importance of wide ranging empirical investigation of formulation options in order to optimize freeze-drying outcomes for biologics.

## Introduction

Freeze-drying is widely used to stabilize labile biological medicines and diagnostics; however, formulation can be critical to ensure maximal preservation of activity and stability (Hubbard et al., 2007). Formulation choices also influence the success of the freeze-drying process and can greatly influence the length and hence affordability of freeze-drying processes (Gervasi et al., 2019; Haueser et al., 2020). Many articles have described the selection processes, and general guidelines have been published (Carpenter et al., 1997; Carpenter et al., 2002; Liu and Zhou, 2021). Typically, ionic salts should be avoided as they reduce the shelf temperature at which drying can be successfully undertaken and indeed make operating conditions impractical (Franks and Auffret, 2007). The choice of the stabilizer has also been well-described, with non-reducing disaccharides being the stabilizer of choice. Other components such as amino acids and surfactants may have an important role to play, and buffer selection may influence activity recovery during the freezing stages.

Lyophilization is also applied commonly in the stabilization of reference materials such as those prepared at the NIBSC on behalf of the World Health Organization. Such standards are physical reference materials, with defined assigned bioactivity (Coxon et al., 2019) established after multiple collaborator evaluation, and standardize and assure the validity of measurements made in many areas of clinical diagnosis and medicine. So, it is critical that they remain stable for their entire lifetimes, often well in excess of a decade. For instance, the international standard for interleukin-2 showed highly preserved bioactivity when tested even after 25 years from preparation (Wadhwa et al., 2013).

Originally discovered as a B cell stimulatory factor (BSF-2) based on its ability to stimulate the maturation of B cells into immunoglobulin-secreting cells (Kishimoto and Ishizaka, 1973), human interleukin-6 (IL-6) was isolated and cloned in 1986 (Hirano et al., 1986). Since then, extensive studies have tried to elucidate the structural aspects of IL-6 and its highly complex biology, including its receptors and signaling pathways (e.g., the classical, the trans-signaling and the trans-presentation), given its ability to elicit a diverse array of functions relevant to tissue homeostasis, hematopoiesis, metabolism, and immune regulation (Garbers et al., 2018; Choy et al., 2020). Structurally, IL-6 is a four-helical cytokine of 184 amino acids with two potential N-glycosylation sites and four cysteine residues. The core protein is about 20 kDa, and the glycosylation accounts for the 21-to-26-kDa size of natural IL-6 (Tanaka and Kishimoto, 2014). IL-6 is a pleiotropic cytokine secreted in response to appropriate stimulation during infection, inflammation, or cancer by multiple cell types and exerts both pro- and anti-inflammatory effects, which are of critical importance in regulating B cell and T cell responses and for coordinating the activity of the innate and adaptive immune systems (Tanaka and Kishimoto, 2014). Dysregulation in IL-6 can cause chronic inflammation, autoimmune disorders, and malignancies, and so IL-6 is a key target for clinical intervention with various anti–IL-6/IL-6 receptor therapeutics approved or in development (Garbers et al., 2018; Choy et al., 2020). These include siltuximab, a monoclonal antibody (mAb) targeting IL-6 for use in Castleman’s disease and mAbs such as tocilizumab, which is indicated for use in rheumatoid arthritis (RA), juvenile idiopathic arthritis, adult-onset Still’s disease, giant cell arteritis, Takayasu arteritis, for cytokine release syndrome (CRS) associated with CAR-T cell therapy, and more recently for COVID-19 treatment by the European Medicines Agency and Sarilumab for RA; both mAbs bind and block the IL-6 receptor subunit of the IL-6 receptor (Garbers et al., 2018; Choy et al., 2020; https://www.ema.europa.eu/en/news/ema-recommends-approval-use-roactemra-adults-severe-covid-19).

The International Standards (IS) from the WHO function in value assigning and for controlling the potency of cytokine therapies, where relevant, in standardizing cytokine assays, which may direct and advise therapeutic interventions and also in other applications. For instance, the IL-6 IS is used for calibrating IL-6 preparations which are in use 1) as critical reagents in cell-based assays for potency testing of mAbs-targeting IL-6 and IL-6 receptors, 2) as cell-culture supplements for growth of antibody-producing hybridomas and *ex vivo* expansion of hemopoietic stem cells, and 3) in calibrating immunoassays for measuring IL-6 levels as a biomarker of inflammation or disease pathology in clinical settings, for example, sepsis, autoimmune, infectious diseases, and in nonclinical and/or clinical/safety testing of immunotherapies (e.g., cytokine-related syndrome associated with CAR-T cells).

The first international standard for interleukin-6 (NIBSC code 89/548) was launched in 1992 (Gaines Das and Poole, 1993) and shown to be a suitable standard for such applications. However, stocks are running low after nearly 30 years of use, and an exercise was undertaken in lyophilizing a replacement material, including revisiting the formulation and freeze-drying cycle design to accommodate process advancements and current best practice. Using human interleukin-6 (IL-6) as an example, we emphasize the importance of thermal analytical methods in predicting successful freeze-drying outcomes to provide efficiencies by avoiding expensive and time-consuming unacceptable freeze-drying runs.

## Materials and Methods

### Formulation Options

IL-6 (Bio-Techne, Minneapolis, United States) was formulated to achieve a 1 μg/ml solution post reconstitution of the ampoule, given that the first WHO IS also contains 1 µg per ampoule, and a majority of IL-6 bioassays for the activity of IL-6 often begin at a starting concentration of 1–2 ng/ml. Trehalose was selected as the disaccharide stabilizer since it was used in the production of an earlier interim standard or for the existing first IS (code 89/548). An array of different buffers were selected based on the use of either sodium acetate or sodium phosphate in the formulation of previous IL-6 standards (e.g., 89/548 or 88/514, which served as the interim standard and contained *E. coli*, expressed IL-6 similar to the intended candidate standard prior to the establishment of the first WHO IS code 89/548). Formulations with and without isotonic NaCl were compared in this evaluation.

### Thermal Analysis

Freeze-drying microscopy: analysis was performed using 2-µL aliquots pipetted into a quartz crucible and analyzed using the Lyostat 5 freeze-drying microscope (Biopharma Ltd., Winchester, United Kingdom); with a freeze rate of 10°C/min to −50°C, vacuum was applied and a ramp rate of 5–10°C/min warming was used until the collapse was visible.

Modulated differential scanning calorimetry: (mDSC) (Q2000, TA Instruments, Wilmslow, United Kingdom) High-volume steel pans were filled with samples in duplicate (80 µL) against an empty pan, and the samples were frozen to −90°C and then ramped at 3°C/min to ambient temperature with modulation at 1°C/min.

### Freeze-Drying

Trial freeze-drying was performed on a VirTis Genesis 25EL dryer (Biopharma Process Systems, Winchester, United Kingdom), with bespoke 5-ml type I glass ampoules being used and a fill volume of 1 ml. Ampoules (Schott) and halobutyl closures (13 mm diameter igloo) (West Pharma igloo) were obtained from Adelphi Packaging (Haywards Heath, United Kingdom). Filling was performed manually using a Gilson P1000 pipette (Anachem, Luton, United Kingdom), and the samples were dried using a conservative freeze-drying cycle. Freezing was down to −50°C and then primary drying was at −40°C for 22 hours followed by ramping to 25°C and secondary drying for a minimum of 20 h, all at 30 µbar vacuum. Ampoules were backfilled with low-moisture nitrogen and stoppered *in situ* before removal and flame sealing.

Several trial runs were conducted (Table 1).

1) Trial run SS-879 included a comparison of two formulations without active material
  • 0.6% trehalose, 0.2% human serum albumin (HSA), and 50 mM sodium acetate pH5,
  • 0.6% trehalose, 0.1% HSA, and 50 mM sodium phosphate pH 7,

Ampoules were subjected to freeze-drying over 2 days, with a temperature of −40°C, 30 µ bar vacuum primary drying, and 25°C secondary drying. Ampoules from both formulations were analyzed by FDM and mDSC.

2) In trial run SS-880, the successful NaP formulation from SS-879 was scaled up and 60 ampoules dried with 1 μg/ml IL-6 was added. A 4-day instead of a 2-day freeze-drying cycle for SS-879 was used with −40°C primary drying and elevated 30°C secondary drying for 20 h.
3) In trial run SS-889, a comparison was made with both excipients only (without IL-6) and with IL-6 containing formulations, with and without the addition of 0.9% NaCl to the formulation:

These were freeze-dried using a 3-day cycle but with an elevated primary drying shelf temperature of −35°C and a vacuum setpoint of 100 µ bar, (70mTorr) with the intention of removing excess water during drying and of getting a lower final residual moisture.

4) Candidate definitive batch 21/308. A definitive fill was performed in the CS100 (Serail, Arguenteil, France) dryer in the CBRM in November 2021 using a 4-day cycle but based around the conditions applied for trial SS-889.

**TABLE 1:** Descriptors of formulation options SS-879, SS-880, SS-889, and 21/308.

	SS-879		SS-880	SS-889				21/308
Identifier	—	—	—	Red ring	Blue ring	Black stripe	Clear	—
Trehalose	0.6%	0.6%	0.6%	0.6%	0.6%	0.6%	0.6%	0.6%
HSA	0.2%	0.1%	0.1%	0.1%	0.1%	0.1%	0.1%	0.1%
Buffer	50 Mm NaAc pH 5	50 mM NaP pH 7	50 mM Na P pH 7	50 mM Na P pH 7	50 mM Na P pH 7	50 mM Na P pH 7	50 mM Na P pH 7	50 mM Na P pH 7
IL-6	None	None	1 μg/ml	1 μg/ml	1 μg/ml	—	—	1 μg/ml
NaCl	—	—	—	—	0.9% w/v	—	0.9% w/v	0.9% w/v

### Scanning Electron Microscopy

The samples were prepared in a dry box operating at less than 8% humidity (Deben, Bury St Edmunds, United Kingdom) by breaking the glass vial to release the freeze-dried cake. The cake was then sectioned across the approximate center with a razor blade, and the section was removed from the vial. The section was trimmed to produce a ∼5-mm section from the central region, and these were attached to a 12.5-mm SEM stub by Silver Conductive Adhesive 503 (Agar Scientific Stansted, United Kingdom and Electron Microscopy Sciences, Hatfield, PA, United States, respectively), with the “center” side facing upward. Arrows on the stub were used to indicate the top of the cake. When the paint was completely dry, the samples were thinned and leveled with a razor blade, if required, and then sputter-coated with 4 nm gold by using a Leica ACE600 coater (Leica Microsystems, Milton Keynes). The samples were held under vacuum in the sputter coater until they were transferred to the SEM holder. When placed in the holder, the top of the cake was positioned to the posterior of the chamber to allow easy orientation in the microscope. The samples were imaged in a JSM7401F scanning electron microscope (Jeol UK, Welwyn Garden City, United Kingdom) at 5 kV, 1.5–3 µA, with a probe current of 7. The images were acquired by the in-lens secondary electron detector (LEI) at magnifications of ×300, ×850, and ×1,500 as required. For each cake, three positions were imaged at the bottom of the cake, the center of the cake, and the top of the cake.

**Residual moisture** was measured using Karl Fischer coulometric titration on an automated CA-200 Mitsubishi coulometer with a Gilson GX-270 robotic sampler (A1 Envirosciences, Blyth, United Kingdom). Freeze-dried samples were broken up in a pyramid dry bag (Cole Parmer, London, United Kingdom) under low moisture conditions (∼10% RH maintained by a continuous flow of dry nitrogen) and then dispensed into HPLC autosampler vials (4-ml screw-capped vials fitted with PTFE membrane pierceable lids, C4015-88 Thermo Scientific, Hemel Hempstead, United Kingdom). The samples were weighed and analyzed in triplicate, and the coulometer calibration was checked with bracketed samples of a water standard (Mitsubishi Aquamicon P, A1 Envirosciences). Container blanks (containing no product) were also run and subtracted from the observed moisture content. Moisture on the definitive batch was determined by manual coulometric Karl Fischer titration on the same model coulometer but operated in a nitrogen dry box.

**Oxygen Headspace content:** Ampoules were backfilled with nitrogen prior to sealing, and so as an indicator of the integrity of the sealed ampoules, measurements of the oxygen headspace content were made non-invasively using infrared frequency–modulated spectroscopy at 760 nm (for oxygen) with the FMS-760 spectrometer (Lighthouse Instruments, Charlottesville VA, United States) against NIST traceable oxygen standards in identical container types.

### Bioactivity Assay

The bioactivity of IL-6 preparations was measured using the human embryonic kidney 293 (HEK293)–derived HEK-Blue™ IL-6 reporter gene cell line (Invivogen, Toulouse, France), stably transfected with genes encoding human IL-6R and STAT3 linked to a reporter gene expressing secreted embryonic alkaline phosphatase (SEAP) under the control of the IFN-β minimal promoter. The cell line was cultured, and the bioassay was performed as per the manufacturer’s procedure. Briefly, IL-6 was serially diluted (from 2000 pg/ml to 1.95 pg/ml) in assay medium (Dulbecco’s modified Eagle medium containing 10% heat-inactivated fetal bovine serum, 2 mM L-glutamine, and penicillin/streptomycin (50U/ml/50 μg/ml) in 96-well tissue culture plates, and 5 × 10^4^ cells were added to individual wells. Following incubation for 20–24 h at 37°C in a humidified CO_2_ incubator, SEAP levels were measured by incubating cell supernatants with QUANTI-Blue™ solution (Invivogen, Toulouse, France) as per the manufacturer’s instructions for 2 h at 37°C in a humidified CO_2_ incubator. The quantity of SEAP secreted into the cell supernatant is proportional to the IL-6 concentration and determined by measuring the absorbance at 620 nm using a microplate reader (Spectramax M5, Molecular Devices UK, Wokingham, United Kingdom). Estimates of potency relative to the bulk unformulated IL-6 were calculated using CombiStats v6.0 (www.edqm.eu), with a sigmoid curve model.

## Results

### Freeze-Drying Appearance

**FIGURE 1:**
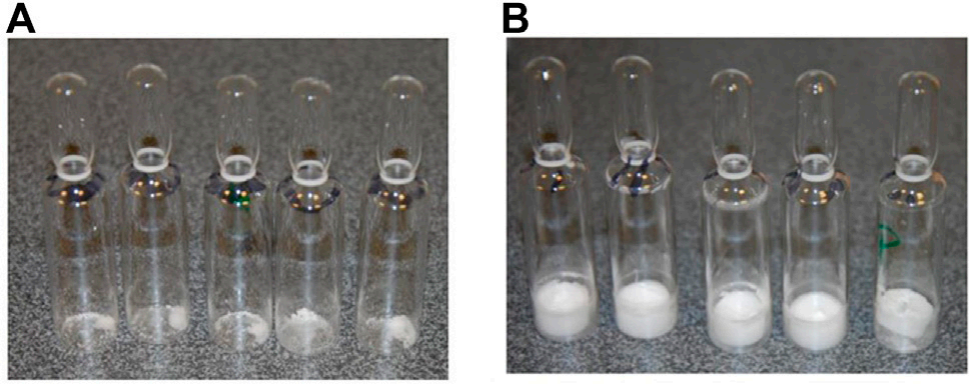
FD trial SS-879 options **(A)** acetate and **(B)** phosphate-physical appearance.

Both acetate- and phosphate-based formulations (SS-879) were compared in the first trial (Figure 1) and under the conditions used revealed formation of a good cake for the NaP buffer but a total collapse and powder-like appearance of the lyophilizate with the formulation containing acetate buffer under these quite conservative drying conditions. So, phosphate was subjected to a repeat trial (SS-880) and again performed well. The bioactivity was good, but the residual moisture content was high >2% despite an extended period of secondary drying. An increase in the duration of the freeze-drying cycle to 4 days failed to provide an acceptable moisture content (<1%w/w).

**FIGURE 2:**
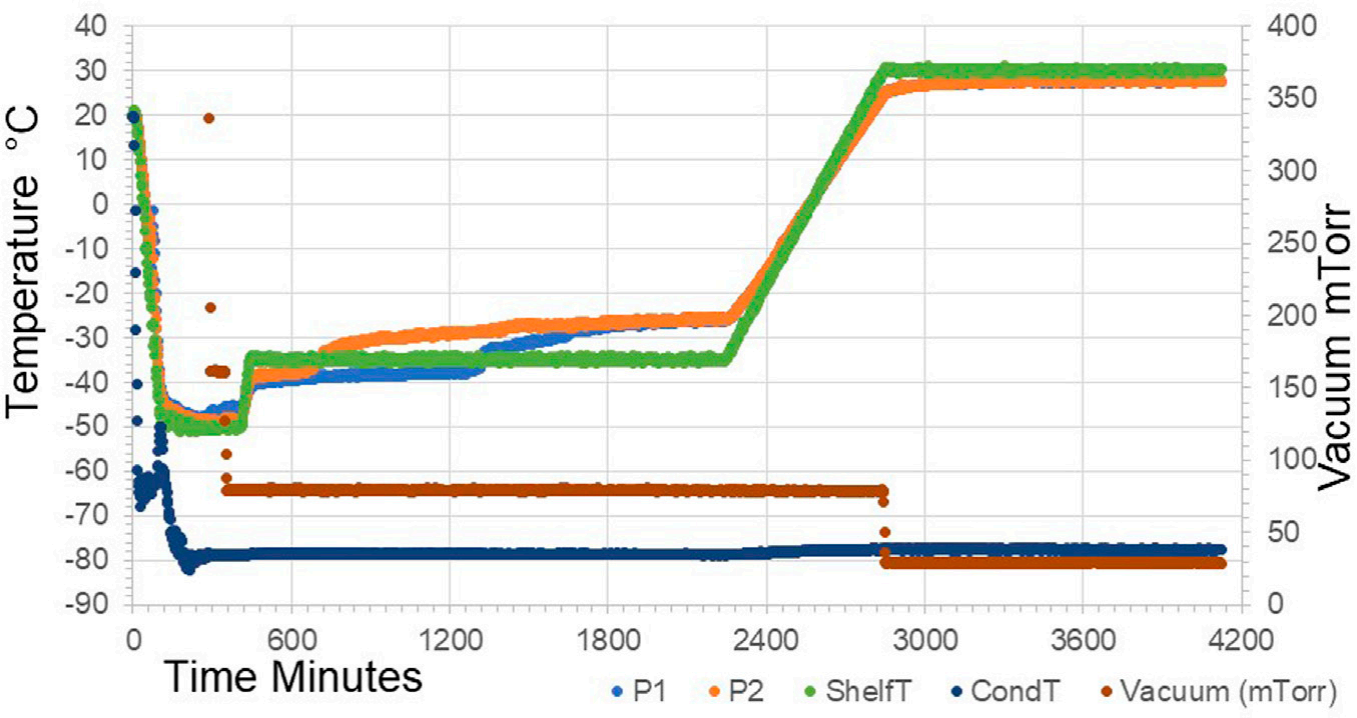
Freeze-drying profile of SS-889 trial with (P1) and without (P2) additional NaCl in formulation.

**FIGURE 3:**
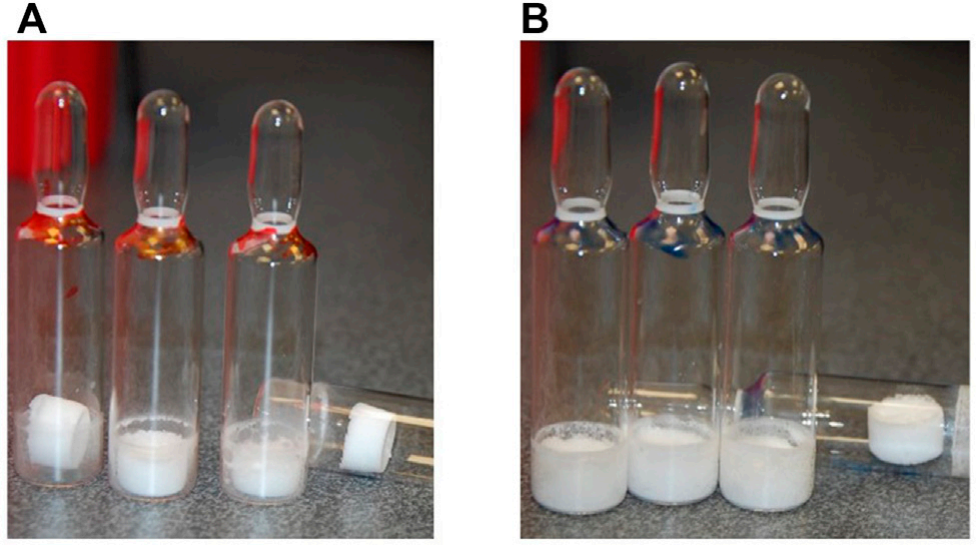
SS-889 FD options **(A)** without NaCl and **(B)** with NaCl (SS-889).

The previous interim standard with *E. coli–*expressed IL-6 (coded 88/514) had been formulated in a base of saline, and hence 0.9 w/w NaCl was added in to the formulation and compared (trial SS-889, Figure 2) with the low salt formulation (NaCl-free). Surprisingly, although both gave good freeze-dried cakes, Figure 3 the moisture of the isotonic NaCl formulation was much lower on the same cycle than the formulation without NaCl, both in excipient-only ampoules and those formulated with active IL-6 (Table 2).

**TABLE 2:** QC batch details for trials and candidate definitive batch 21/308.

Parameter	Samples of SS-889 trial				—
—	IL-6 no NaCl (red)	IL-6 + NaCl (blue)	Excipient–no NaCl (black stripe)	Excipient + NaCl (clear)	Definitive 21/308
Fill weight g (CV, n_)	0.959 (0.85%, 3)	0.967 (2.38%, 3)	0.939 (7.5%, 3)	0.968 (1.65%, 3)	1.0077 g (CV 0.26%, 183)
Dry weight mg (CV, n)	13.6 (1.27%, 3)	21.3 (3.34%, 3)	13.2 (15%, 3)	21.3 (1.6%, 3)	15.99 mg (CV = 11.8%, 5)
Residual moisture- w/w (CV, n)	2.37% (9.1%, 3)	0.31% (23.7%, 3)	4.3% (35.1%, 3)	0.40% (47.5%, 3)	0.27% (CV = 17.8%, 12)
Appearance	Slight lateral pinching	Adherent	Slight lateral pinching	Adherent	Adherent

A scaled-up candidate definitive batch 21/308 was produced with the isotonic saline formulation and over 5,000 ampoules dried in a Serail CS-100 freeze-dryer (Serail Arguentil, France) with the same cycle. As for SS-889, the isotonic saline formulation resulted in a batch of ampoules with well-formed cakes and low moisture content of 0.27% w/w, with good bioactivity.

### Thermal Analysis

The three formulations, acetate-based, phosphate-based, and phosphate-based plus NaCl, were analyzed by thermal analysis. The acetate-based formulation did not show any clear thermal events, and on drying an unacceptable appearance resulted (Figure1) Freeze-drying microscopy indicated a collapse temperature approximately −33°C (*n* = 2) for the phosphate buffer, but collapse was visible even from below −50°C for the acetate formulation.

**FIGURE 4:**
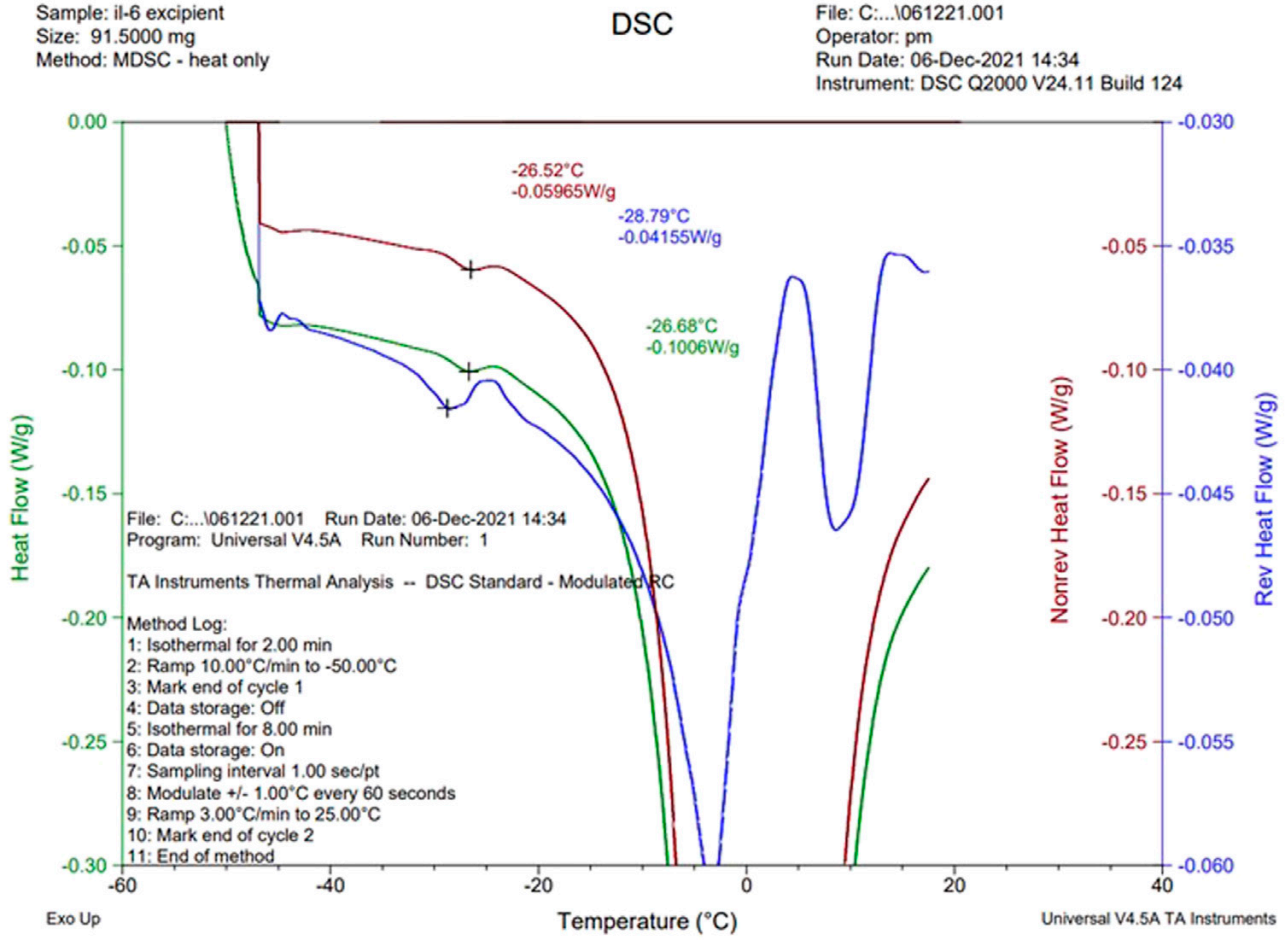
Thermal analysis (mDSC) of sodium phosphate formulation with 0.9% w/v NaCl.

**FIGURE 5:**
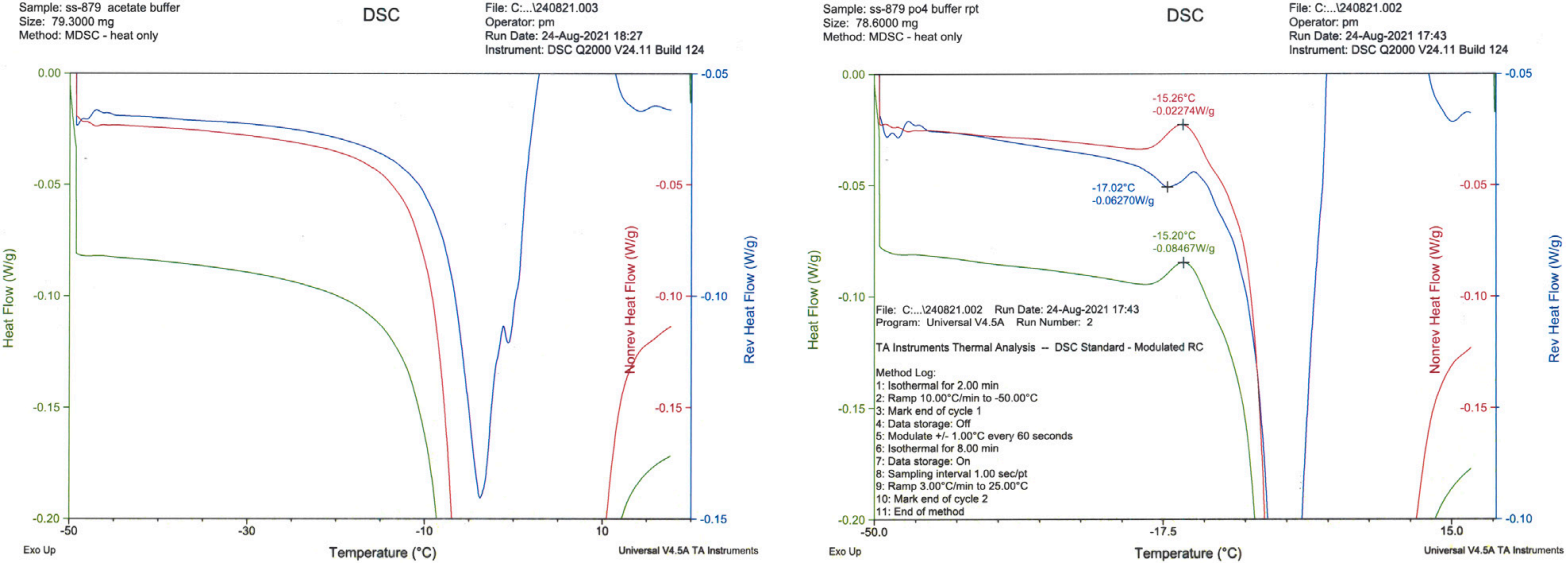
mDSC traces of sodium acetate (LHS) and sodium phosphate (RHS) formulations SS-879.

The NaCl-containing phosphate-buffered excipient and the NaCl-containing definitive product showed a clear event in the mDSC profile at approximately −26°C, indicative of crystallization of NaCl (Figure 4). This was absent in the NaCl-free formulation, though an exothermic event did occur around −15°C (Figure 5).

**FIGURE 6:**
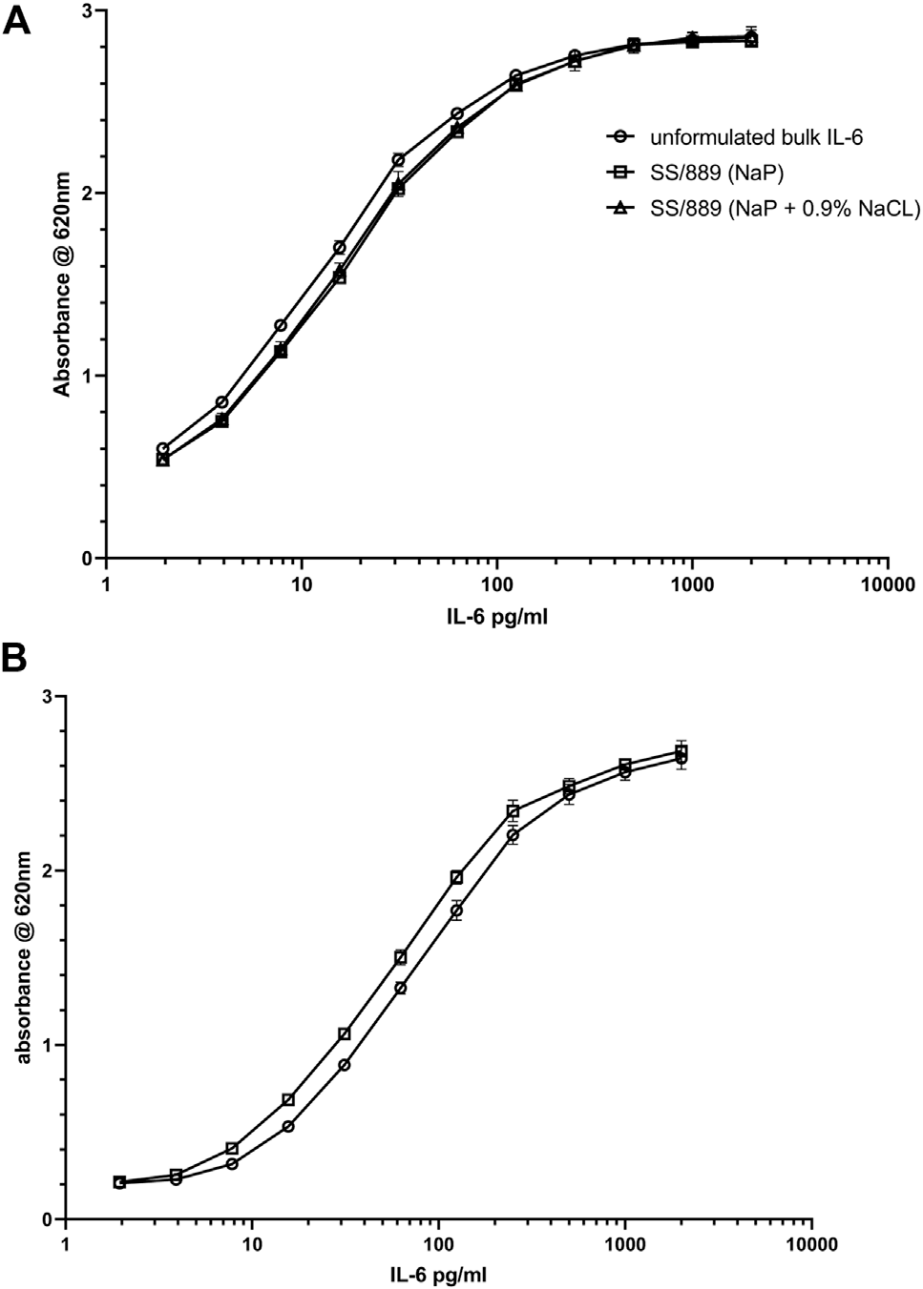
Bioactivity of IL-6 samples **(A)** (IL-6 trial SS-889) in NaP formulation with (open triangle) and without (open square) addition of 0.9% NaCl, compared with IL-6 unformulated bulk material (open circle) in the HEK-Blue™ IL-6 reporter gene assay and **(B)** bioactivity of the IL-6 candidate preparation 21/308 (open square) compared to the international standard IL-6 89/548 (open circle) in the HEK-Blue™ IL-6 reporter gene assay.

**Bioactivity data**: Evaluation of the bioactivity of the formulations with and without NaCl showed acceptable retention of bioactivity, if anything slightly higher for the formulation with NaCl (78 vs. 73% over six measurements) as shown in Table 3. The plots of the dilution curves show that the bioactivity of the two formulated preparations was very similar (Figure 6A). The recovery of bioactivity in the candidate definitive batch 21/308 containing NaCl in the formulation was also well-preserved (Figure 6B) and was comparable to the existing international standard.

**TABLE 3:** Potency estimates for IL-6 SS-889 trial run relative to unformulated bulk IL-6.

Sample	Estimated potency (n)	Lower 95 (%)limit	Upper 95 (%)limit
Lyophilized (NaP)	72.7% (6)	69.7	75.8
Lyophilized (NaP + 0.9% NaCl)	78.0% (6)	74.8	81.3

(n), denotes number of estimates made.

### Structural Analysis by SEM

**FIGURE 7:**
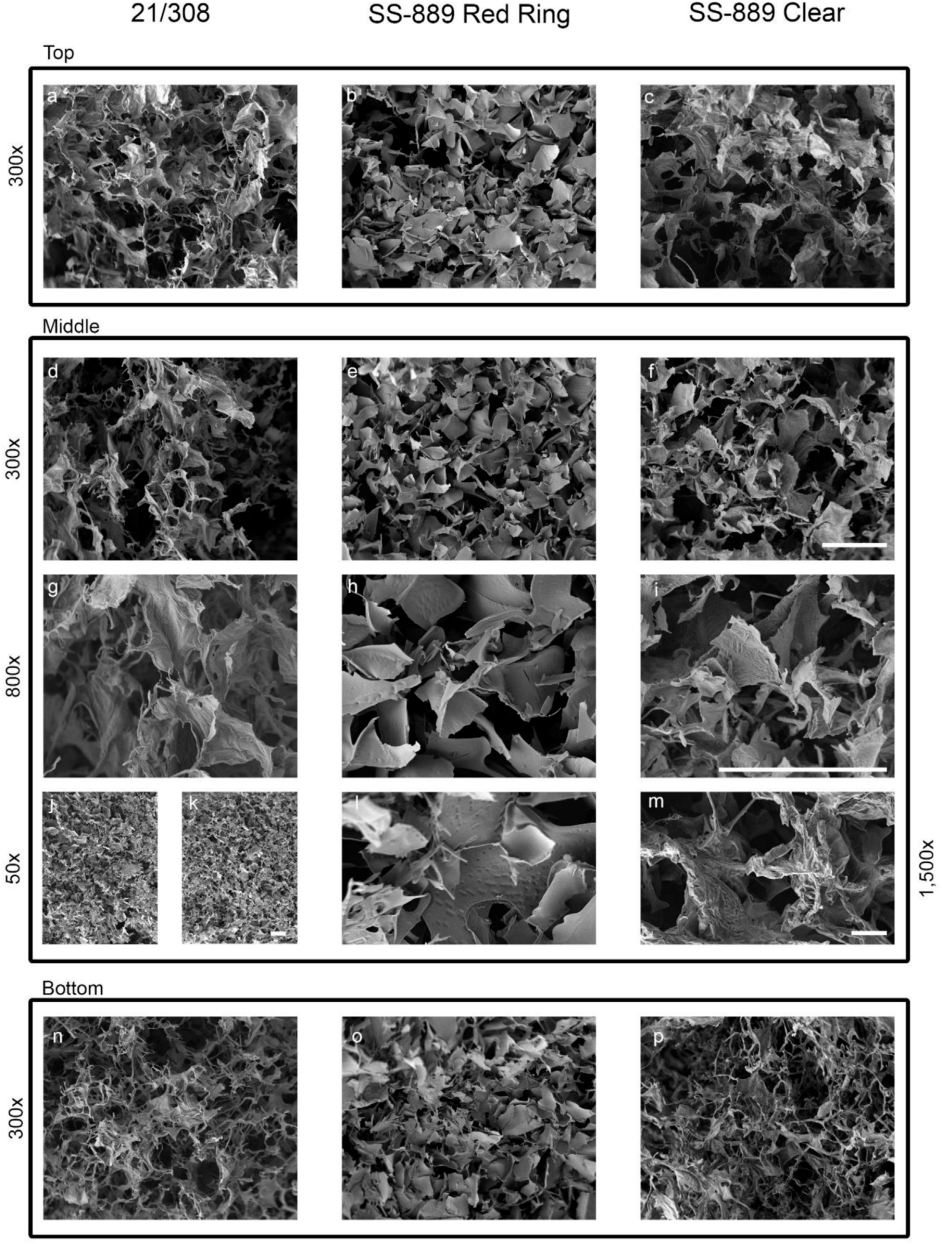
High-resolution SEM images of the NaCl-containing definitive (21/308) and the with NaCl (SS-889 clear) and without NaCl (SS-889 red ring) trial freeze-dried cakes. Images a-f and n-p show top, middle and bottom of the cakes at 300 × magniifcation. Images g-i show a highr magnificiation view at 800× of the middle section of the cakes. Images j to m show lower ×50 (j,k) and higher ×1,500 magnifications [(l,m), respectively] of formulations without NaCl, (j) and (l) (SS-889 red ring), and with NaCl, (k) and (m) (SS-889 clear). Gradation scale = 100 µm in all panels.

SEM was undertaken on NaCl-omitted and NaCl-containing formulations and also on the candidate definitive batch 21/308 (Figure 7). Examination of the freeze-dried cakes of formulations with and without NaCl showed that NaCl-containing formulation (SS-889 clear and 21/308) (Image J) had a more consistent and well-formed cake with a wider open-pore structure than that formulated without NaCl (SS-889 red ring) (image k). At the highest resolution, (images g–i and l–m) fine details indicative of a more crystalline structure were evident in NaCl-containing preparations (Figure 7). This is consistent with the NaCl-containing formulated freeze-dried cakes having a more open cake and therefore being able to dry more completely.

## Discussion

For lyophilized materials intended as standards, collapsed appearance is generally unacceptable, although this may not necessarily correlate with loss of activity of the material. In general, the advice is to reduce or omit inorganic salts from formulations as they reduce the glass transition temperature value of formulations and result in lower temperature primary drying and hence longer, slower cycles. However, sometimes, the benefits of crystalline excipient can be overlooked. Here, we demonstrated that in terms of the final residual moisture and the cake structure of the freeze-dried mass, NaCl addition provided a crystalline matrix, which resulted in a better pore structure and led to lower residual moisture than a non-NaCl formulation on the same cycle. Most telling is that even though a long and higher temperature secondary drying was used in trial SS-880, the NaCl-free formulation still retained much higher levels of residual moisture, a conclusion supported by the direct comparative study SS-889. Comparative analysis of the two formulations by SEM showed the cake structure with the inclusion of NaCl to be superior, with an open pore structure, and so it is tempting to relate this to the observed poorer structure to the less efficient water sublimation and higher residual moisture content.

This result complements our earlier finding (Duru et al., 2015), which showed that for freeze-dried influenza antigen reference preparations, NaCl crystallization was a critical factor—an increase in the amorphous content using raised levels of sucrose (above 1.4% w/w) resulted in a collapsed freeze-dried appearance. From the mDSC, it was concluded that raising the sucrose content (although usually a desirable stabilizer for freeze drying), resulted in inhibition of the crystallization of the NaCl co-formulant and that this crystallization was critical in producing an acceptable freeze-dried appearance. In the present study also, the presence on mDSC of the eutectic event in the “with NaCl” formulation and its absence in the “without NaCl” sample when analyzed correlated to the crystallization of the NaCl, resulting in improved overall structure of the freeze-dried cake.

Shalaev et al. (1996) described the glass transitions, softening points, devitrification, and melting points for the ternary system water−sucrose−sodium chloride by DSC and showed that NaCl crystallization can be inhibited by amorphous solutes such as sucrose. Goshima et al. (2016) have shown that inclusion of NaCl may improve the stability of freeze-dried model protein albumin. Thakral et al. (2021) have systematically reviewed the interactions between formulation components both in frozen and freeze-dried systems.

In conclusion, from the IL-6 standard perspective, the addition of NaCl to the NaP formulation provided a robust cake with a lower moisture content below 1% w/w, which in agreement with the WHO specifications, is more likely to give a highly stable reference material (WHO, 2006). The difference in formulation did not seem to have any negative influence on the bioactivity as measured by the reporter gene assay. The NaCl-containing formulation was, therefore, selected for scale up for the candidate definitive material 21/308.

More generally, in terms of formulation for freeze-drying, though guided by general principles (Kharaghani et al., 2017), consideration must be given on a case-by-case basis to the influence of individual components on the thermal properties and final freeze-dried appearance of any given formulation. This again underlines the value of thermal analytical methods in predicting successful freeze-drying outcomes and other new analytical techniques are being introduced (Thomik et al., 2022), and their use will help avoid expensive and time-consuming unacceptable freeze-drying runs in the development of lyophilization processes.

## Data Availability

The datasets presented in this article are not readily available because none of them are mentioned. Requests to access the datasets should be directed to paul.matejtschuk@nibsc.org.
