# Erratum: Impact of formulation choices on the freeze-drying of an interleukin-6 reference material

**DOI:** 10.3389/fmolb.2022.1062810

**Published:** 2022-10-19

**Authors:** Frontiers Production Office

**Keywords:** freeze drying, formulation, interleukin-6, sodium chloride, scanning electron microscopy, differential scanning calorimetry

Due to a production error, the reference for “Coxon et al., 2019” was incorrectly written as “Longstaff, C., and Burns, C. H. (2019). Applying the Science of Measurement to Biology: Why Bother? PLoS Biol. 17 (6), e3000338. doi:10.1371/journal.pbio.3000338.” It should be “Coxon CH, Longstaff C, Burns C (2019). Applying the science of measurement to biology: Why bother? PLoS Biol 17(6), e3000338. doi:10.1371/journal.pbio.3000338.”

Also, there was an error in a citation. In the introduction section, instead of “Coxon et al., 2021” it should be “Liu and Zhou, 2021” and the reference was incorrectly written as “Coxon, C., Longstaff, C., and Burns, C. (2021). Freeze-Drying of Proteins. Methods Mol. Biol. 2180, 683–702. doi:10.1007/978-1-0716-0783-1_37.” It should be “Liu, B., and Zhou, X. (2021). Freeze-drying of proteins. In: Cryopreservation and Freeze-Drying Protocols. Editors W. F. Wolkers and H. Oldenhof (New York, NY: Humana), 2180. 10.1007/978-1-0716-0783-1_37.”

The publisher apologizes for this mistake. The original version of this article has been updated.
